# The Transient Sea Level Response to External Forcing in CMIP6 Models

**DOI:** 10.1029/2022EF002696

**Published:** 2022-10-24

**Authors:** Aslak Grinsted, Jonathan Bamber, Rory Bingham, Sammie Buzzard, Isabel Nias, Kelvin Ng, Jennifer Weeks

**Affiliations:** ^1^ Physics of Ice, Climate, and Earth Niels Bohr Institute University of Copenhagen Copenhagen Denmark; ^2^ School of Geographical Sciences University of Bristol Bristol UK; ^3^ Department of Aerospace and Geodesy Data Science in Earth Observation Technical University of Munich Munich Germany; ^4^ School of Earth and Environmental Sciences Cardiff University Cardiff UK; ^5^ School of Environmental Sciences University of Liverpool Liverpool UK; ^6^ School of Geography, Earth and Environmental Sciences University of Birmingham Birmingham UK; ^7^ Met Office Exeter UK

**Keywords:** sea level rise, ice loss, thermosteric, model validation, sensitivity, projections

## Abstract

Earth is warming and sea levels are rising as land‐based ice is lost to melt, and oceans expand due to accumulation of heat. The pace of ice loss and steric expansion is linked to the intensity of warming. How much faster sea level will rise as climate warms is, however, highly uncertain and difficult to model. Here, we quantify the transient sea level sensitivity of the sea level budget in both models and observations. Models show little change in sensitivity to warming between the first and second half of the twenty‐first century for most contributors. The exception is glaciers and ice caps (GIC) that have a greater sensitivity pre‐2050 (2.8 ± 0.4 mm/yr/K) compared to later (0.7 ± 0.1 mm/yr/K). We attribute this change to the short response time of glaciers and their changing area over time. Model sensitivities of steric expansion (1.5 ± 0.2 mm/yr/K), and Greenland Ice Sheet mass loss (0.8 ± 0.2 mm/yr/K) are greater than, but still compatible with, corresponding estimates from historical data (1.4 ± 0.5 and 0.4 ± 0.2 mm/yr/K). Antarctic Ice Sheet (AIS) models tends to show lower rates of sea level rise (SLR) with warming (−0.0 ± 0.3 mm/yr/K) in contrast to historical estimates (0.4 ± 0.2 mm/yr/K). This apparent low bias in AIS sensitivity is only partly able to account for a similar low bias identified in the sensitivity of global mean sea level excluding GIC (3.1 ± 0.4 vs. 2.3 ± 0.4 mm/yr/K). The balance temperature, where SLR is zero, lies close to the pre‐industrial value, implying that SLR can only be mitigated by substantial global cooling.

## Key Points

- The rate of ice sheet mass loss and ocean expansion has a near linear relationship to global mean surface warming in both models and data
- The modeled transient sensitivity of the sea level budget to warming is compatible with historical behavior except for Antarctic mass loss
- Models of Antarctic mass loss may be biased as data shows increasing losses with warming in contrast to models

## 1 Introduction

Despite recent advances in both observations and numerical modeling, sea level rise (SLR) projections remain highly uncertain due, in large part, to inadequate understanding of how the ice sheets covering Antarctica and Greenland will respond to climate forcing. Various approaches have been developed to attempt to address this uncertainty including community‐based model intercomparison projects (Goelzer et al., 2020; Nowicki et al., 2016; Seroussi et al., 2020), emulator studies (Edwards et al., 2021), structured expert judgment (Bamber et al., 2019; Bamber & Aspinall, 2013) and, what have been termed, semi‐empirical models (SEMs). This latter approach correlates changes in global surface temperature with global mean sea level (GMSL) based on how these two variables have evolved in the past (Moore et al., 2013). Various approaches have been used to account for the different time constants for the response of the components of the climate system that contribute to SLR, such as thermal expansion of the oceans, and mass loss from glaciers and ice caps and the Greenland and Antarctic ice sheets. The different approaches mentioned above have produced markedly different estimates for future SLR, especially for the upper tail of the distributions and depending on the climate forcing scenario used (Fox‐Kemper et al., 2021). The ice sheets have the longest response time of any component of the climate system and their behavior for a given year does not, therefore, reflect the climate forcing for that year, or preceding decade but the cumulative forcing over a longer time period. To account for this delayed response and to provide a scenario‐independent metric for SLR, Grinsted and Christensen developed the concept of the transient sea level sensitivity (TSLS) (Grinsted & Christensen, 2021). This is analogous to the notion of a transient climate sensitivity, which defines the mean temperature response of a GCM to a doubling in CO_2_ concentrations. In the case of SLR, however, the initial transient response to warming is a change in the sea level rate due to the slow response of ice sheets and oceans. The TSLS is therefore defined as the instantaneous change in rate of SLR associated with a change in temperature (Grinsted & Christensen, 2021). The ice sheets and oceans respond slowly to warming and the response over a century can be considered transient. In practice, the TSLS has therefore been estimated using a century‐average temperature to determine the transient sea level response at the end of the 100‐year period (Grinsted & Christensen, 2021). This is a useful metric because it is (a) scenario independent and (b) it is not the equilibrium SLR that is critical for adaptation planning but the rate over some time period (Oppenheimer et al., 2019).

An important conclusion of the study that introduced the concept of the TSLS was that projections for SLR presented in the Fifth Assessment Report (AR5) of the IPCC and their Special Report on Oceans Cryosphere and Climate (SROCC) had weaker sensitivities than indicated by the observational and proxy sea level record (Grinsted & Christensen, 2021). This suggests that the model‐based projections in the AR5 and SROCC likely underestimate future SLR when compared to observations. This conclusion is supported by studies using structured expert judgment (Bamber et al., 2019) and from simple extrapolation of the present‐day, forced SLR trends from satellite altimeter observations (Nerem et al., 2022). The difference was significant and, assuming a linear relationship between TSLS and the centennial‐average temperature change, the observations lie closer to expert judgment median projections than to the numerical model estimates. This is likely due to smaller ice sheet contributions from the numerical models although an investigation of the sensitivity of each component of the sea level budget was not undertaken (Grinsted & Christensen, 2021). This raises two interesting and important questions. First, there is evidence suggesting that CMIP6 models have a higher climate sensitivity compared to their predecessors (Forster et al., 2020) and, indeed, this has lead to the conclusion that the Greenland Ice Sheet (GrIS) produces a larger contribution to SLR compared to their predecessors when forced by these models (Hofer et al., 2020) and that the steric contribution is about 10% greater than in CMIP5 (Jevrejeva et al., 2020). It is possible, therefore, that the TSLS for CMIP6 simulations lies closer to the observational trend relative to CMIP5 and the AR5 values. Second, data are available for each component of the sea level budget for CMIP6 simulations making it possible to examine the transient sensitivity of each of these and to compare them with whatever suitable observational data are available. This permits identification of which modeled components are less sensitive with respect to observations and to quantitatively assess the origin for the discrepancy between the observations and modeled TSLS identified in Grinsted & Christensen, 2021. Those two questions are addressed in this study: namely an examination of the TSLS of the GrIS, West and East Antarctic Ice Sheets (WAIS, EAIS), glaciers and ice caps (GIC) and ocean thermal expansion separately based on the CMIP6 model runs used in the Sixth Assessment Report (AR6) of the IPCC (Fox‐Kemper et al., 2021) and an evaluation of the CMIP6 TSLS against observations and previous modeled SLR trends.

## 2 Data

### 2.1 Ice and Ocean Observations and Paleo Sea Level Proxies

The observations and their errors are based on the assessment of the historical sea level budget in AR6 (Fox‐Kemper et al., 2021). From this we extract the rates in four distinct periods (1901–1970, 1971–1992, 1993–2005, 2006–2018) for the steric, Antarctic Ice Sheet (AIS), GrIS, and GIC contributors and for GMSL. The AR6 represents a synthesis of all relevant knowledge, but the data sources are similar to a recent study that demonstrated closure of the sea level budget components when compared to the integral as inferred from sea surface height data (Frederikse et al., 2020). This study included satellite and in situ assessments starting in 1960 for GIC, 1970s for the GrIS and 1992 for the AIS. Modeled and/or observational estimates for both the GrIS and GIC extend back to 1900 but not for the AIS. Several lines of evidence suggest some mass loss from the Antarctic Peninsula (PEN) and WAIS but a relatively stable (EAIS) (Adhikari et al., 2018). These older (pre satellite) observations, however, have a significantly larger uncertainty associated with them. The thermosteric component of the sea level budget was obtained from three different compilations of in situ observations from 1957 to 2018 combined with a reconstruction based on more limited historical data extending back to 1871 (Zanna et al., 2019). Thus we use reconstructed observational estimates from 1901 to 2018 for all components except the AIS. These are supplemented with estimates for the 1850–1900 pre‐industrial period for GMSL and GIC. GMSL rose by 0.5 ± 0.2 mm/yr between 1850 and 1900, according to a global synthesis of regional sea‐level reconstructions spanning the last 3 millenia (Gulev et al., 2021; Kemp et al., 2018; Kopp et al., 2016). The GIC contribution for 1850–1900 was estimated by Marzeion et al. (2015) based on an upscaling of observed glacier change to a global inventory of glaciers. This does not include mass lost from glaciers missing from the inventory. Parkes and Marzeion (2018) estimate that these missing glaciers account for 25%–50% of the mass change in the early part of the twentieth century. We therefore multiply the Marzeion et al. (2015) estimate with 1.6 (the geometric mean of the upper and lower bound). We interpret the Parkes & Marzeion, 2018 upper/lower bound as a ± 2*σ* range. This results in a GIC estimate for 1850–1900 of 0.7 ± 0.2 mm/yr. The AR6 only reports the historical contribution for the entire Antarctic ice sheet, and we therefore supplement with estimates for the WAIS, EAIS, and PEN from IMBIE2 (Shepherd et al., 2018) for the period from 1992 to 2017. The observations of sea level rates are paired with corresponding estimates of Global Mean Surface Temperature (GSMT) based on HadCRUT5 (Morice et al., 2021). Throughout, we report temperatures as anomalies relative to a 1995–2014 baseline for both observations and models.

### 2.2 CMIP6 Data

The sixth phase of the Coupled Model Intercomparison Project (CMIP6) brings together an advanced set of participating climate models compared to CMIP5. CMIP6 models were forced by an updated set of emissions scenarios, utilizing the Shared Socioeconomic Pathways (SSPs), creating a broader selection of possible futures. For the first time, CMIP6 included an ice sheet modeling intercomparison (Goelzer et al., 2020; Nowicki et al., 2016; Seroussi et al., 2020) and experiments investigated the effects of higher ocean resolution (e.g., HighResMIP). Since CMIP5, greater understanding of physical processes (e.g., glacier and ice‐shelf calving and grounding line evolution) have driven developments in glacier and ice sheet models, and the representation of ocean processes has improved with increased resolution (e.g., ocean eddies in a number of models). The closure of the global energy and ocean mass budget after removing drift has also improved in CMIP6 (Irving et al., 2021).

Studies have found that some CMIP6 GCMs have higher equilibrium climate sensitivity compared to CMIP5 (Forster et al., 2020), attributed to improved representation of clouds. This translates into higher projections of GMST change (Hermans et al., 2021) and impacts on projections of regional sea‐level change, such as dynamic sea‐level change in the North Atlantic and Arctic (Lyu et al., 2020). The AR6, however, did not rely solely on CMIP6 simulations for projections of sea level change and used emulators, calibrated to an assessed range of climate sensitivity from paleoclimate observations, and physical process models. This reduced the influence of higher warming found in some CMIP6 models.

### 2.3 Steric Model Output

The thermosteric sea‐level change is obtained from either the CMIP6 direct output of global average thermosteric sea level change (the standard output variable conventionally labeled “zostoga”) or calculated based on potential temperature and salinity in CMIP6 output. For historical outputs, the thermosteric sea‐level change time series are divided into four periods which are: 1850–1900, 1900–1950, 1950–2000, and 1992–2014. For the future climate scenario, we have investigated SSP1‐2.6, SSP2‐4.5, and SSP5‐8.5, for two time periods: 2016–2050, and 2051–2100. Table S1 shows the CMIP6 models and variants used in this study. It should be noted that individual model runs could produce negative rates of thermosteric sea‐level change due to model drift. We therefore correct for model drift by applying a constant rate bias adjustment that sets the 1958–2015 steric rate to exactly match an observational estimate of 0.54 mm/yr (Frederikse et al., 2020). This constant rate adjustment does not affect the sensitivity to a change in temperature.

### 2.4 Land Ice Model Output

The ice sheet and GIC contributions to SLR have been the focus of two model intercomparison projects—ISMIP6 and GlacierMIP. Edwards et al. (2021) emulated glacier simulations from GlacierMIP Phase 2 (Marzeion et al., 2020), ensuring any peripheral glacier overlap with ice sheets was minimal. The models in these intercomparison projects were driven by a relatively small subset of CMIP5 (Goelzer et al., 2020; Seroussi et al., 2020) and CMIP6 models (Payne et al., 2021). Edwards et al. (2021) constructed an emulator tuned to reproduce these MIPs, and used this to project ice mass loss for a modern set of scenarios. Temperatures are projected by a reduced complexity climate model (Smith et al., 2018), allowing for uncertainty in climate sensitivity in a manner that approximates the AR6. In this paper we use a published sample of 500 simulations from the emulator. Each sample from the emulator models the contributions from GrIS, GIC, EAIS, WAIS, and the Antarctic Peninsula, and each sample has been run for six different SSP scenarios. The emulator model projections were pre‐processed with a first order Savitzky‐Golay filter with a window length of 15 years to reduce interannual variability. Neither ISMIP6, nor the emulator, has hindcasts that can be used to assess model drift. A constant drift can be considered as an unforced contribution to SLR and therefore does not affect estimates of the transient sensitivity to a temperature change.

Mass loss from the ice sheets can be partitioned into ice dynamic and surface mass balance (SMB) components. To make an assessment as to which of these components is driving the ice sheet's TSLS, we used the output from the ISMIP6 ice sheet models that were forced by CMIP6 models, reported in Payne et al. (2021). The CMIP6 models used to drive the ice sheet models were limited to those available to the ISMIP6 project at the time—these consist of four models for SSP5‐8.5 and one for SSP1‐2.6. The models are all at the upper end of the CMIP6 ensemble in terms of their transient climate sensitivity (Payne et al., 2021). The ice dynamic sea level contribution is calculated by subtracting the SMB, integrated over the grounded ice sheet area, from the total sea level contribution.

## 3 Methods

The major contributors to SLR can be viewed as large reservoirs. The ice sheets and GIC are reservoirs of freshwater, and the ocean is a reservoir of heat. Any change in the stock of these reservoirs will result in a change in sea level. A steady state is characterized by a balance in the fluxes to and from these reservoirs, for example, gains from snow fall must be balanced by losses from melt and discharge. The initial impact of a change in climate will be a shift in the flux balance of every reservoir, and thus a change in the corresponding sea level rates. However, the fluxes to and from reservoirs are not only influenced by external forcing but will also depend on the stock in the reservoir. The long wave radiation losses from the ocean surface depend on sea surface temperature and are thus connected to ocean heat, and total melt losses from GIC depend on the remaining glaciated area. This leads to a feedback between the stock in the reservoir and the net fluxes. The reservoir leaks or gains until it finds a new equilibrium with the imposed climate, and the equilibration process can be characterized by an e‐folding timescale. The ocean and ice sheets are giant reservoirs that change size slowly and have multi‐centennial equilibrium response times. GIC, however, come in many sizes—every glacier or ice cap with its own response time. Often glacier response times are measured in decades.

In this paper, we focus on the century scale response of the primary contributors to the sea level budget. The chosen time frames are relatively short compared to the response times we expect from most contributors. Thus we will be focusing on the initial “transient” response. We use GMST as an indicator for the intensity of the climate forcing, and investigate how sensitive the transient response is to a change in forcing intensity, that is, mean temperature. As we are primarily concerned with changes in the response rather than absolute values, we disregard small quasi‐constant components of the sea level budget such as the effect of ocean bottom deformation (order 0.1 mm/yr) (Vishwakarma et al., 2020), the deep ocean steric term (order 0.1 mm/yr) or land hydrology (order −0.15 mm/yr) (Fox‐Kemper et al., 2021).

The Transient Sea Level Sensitivity (TSLS) is defined as the initial increase in the rate of SLR to an increase in global mean surface air temperature (Grinsted & Christensen, 2021). We write

(1) $$\mathrm{TSLS}\equiv \frac{d\dot{S}}{dT},$$

where *T* is the GMST anomaly, and $\dot{S}$ is the rate of SLR. We aim to estimate the TSLS for each of the major contributors to the sea level budget from both models and observations. The TSLS concept inherently represents a linearization of the response to warming of the form

(2) $$\dot{S}=\mathrm{TSLS}\cdot T+{\dot{S}}_{0},$$

where ${\dot{S}}_{0}$ is the rate of SLR for a zero GSMT anomaly. We can therefore estimate the TSLS from the slope in a linear regression from a data set of *T* and $\dot{S}$. This is an approximation with a limited range of applicability, as discussed in more detail later. GMST is not a perfect representation of the forcing intensity. For example, the Arctic oscillation is associated with substantial year to year variability in Greenland mass loss which is not captured by a global metric such as GMST. This variability can be reduced, and correlation improved, by temporal averaging of both *T* and $\dot{S}$. For TSLS to be considered a “transient” sensitivity we must consider time intervals that are relatively short compared to the equilibration time. Grinsted and Christensen (2021) argued that TSLS is close to stationary on a century time scales due to the large inertia of the oceans and ice sheets. In this paper we test this assumption by determining and comparing the TSLS in three different periods (historical, early twenty‐first century, and late twenty‐first century). Future projections span a range of scenarios with different warming pathways. This allows us to estimate the TSLS by regressing the temporal average GMST against the corresponding average rate of the modeled contribution to SLR. The regression intercept is the sea level rate associated with a temperature anomaly of zero. The intercept can be reformulated in terms of a balance temperature—the temperature change necessary to stop that sea level component from contributing to SLR (Grinsted & Christensen, 2021). For the historical period we only have a single warming pathway, and thus cannot estimate the sensitivity to warming at a particular point in time. We can, however, examine how the sea level contribution has accelerated over time as warming has progressed.

For every model we calculate the temporal average rate of sea level contribution, and the corresponding average GMST in a set of target periods. We have chosen four historical periods (1850–1900, 1900–1950, 1950–2000, and 1992–2014) and two projection periods (2016–2050, and 2051–2100). The steric contribution is based on CMIP6, and thus covers the entire set of target periods. However, the ice emulator only provides estimates for the future contribution. This is a limitation inherited from the ISMIP6 protocol. We require at least three points in every regression, and reject all poorly constrained TSLS estimates with a standard error greater than 3 mm/yr/K. This quality filter is particularly useful for the 2016–2050 period where the GMST for the different scenarios has not yet deviated by much. We use a different set of periods when we estimate the historical TSLS from observations as we are limited by data availability.

In order to estimate the TSLS we regress GMST against the rate in the sea level contribution. We simply use linear least squares regression for model data. However, for observational estimates we use weighted least squares regression as not all data are equally certain. We weight every data point by the inverse of the estimated standard error in the sea level rate. Confidence intervals in the historical TSLS estimates are determined using a Monte Carlo approach where we perturb the estimated rate and temperature according to the reported standard errors. These perturbations are assumed to be independent, and thus we are assuming no error covariance between the estimated rates in different periods. Fully covariant errors in the sea level rates would only affect the estimated intercept but not the slope. We therefore argue that this assumption has minimal impact on the estimated TSLS confidence intervals.

The spread between CMIP6 runs should not be interpreted as representative of the uncertainty distribution. Some earth system models have been run multiple times, and this can lead to a bias if all CMIP6 runs are treated as equally probable samples from an uncertainty distribution. We therefore average all the model runs from an individual Earth System Model (ESM), with the only exception being those models with perturbed physics which are treated as if it was a different ESM. In this study, CanESM5 is the only model with two different perturbed physics members. Given that there exists substantial difference between different perturbed physics members of CanESM5 in volume‐averaged ocean temperature (Swart et al., 2019), it is sensible to treat it as two different ESMs. The approach we use here is inline with the standard practice of combining multi‐model climate ensembles (Knutti et al., 2010).

There is not a simple one‐to‐one relationship between the ESMs used for the steric model, and the model samples from the ice emulator. It is therefore not trivial to produce a fully consistent model estimate of GMSL. However, it is clear that at the very least we must ensure that only models with similar climate sensitivity are paired. We therefore pair each ice emulator sample (*i*) with the steric estimate from a random sample from the CMIP6 ensemble where each run (*j*) has been assigned a probability weight. The weight, *w*, is designed to account for how well temperatures match. We write

(3) $$w_{ij}=e^{-\frac{1}{2}\;{\left(\frac{T_{i}-T_{j}}{0.2\;K}\right)}^{2}},$$

where the 0.2K is a standard deviation to allow for a small misfit between the two temperatures. This is necessary as we are dealing with finite samples and is similar to the bin width in a histogram. A randomly selected model run (*m*) based on these weights therefore has a small temperature misfit. We make a first order adjustment to the steric rate (${\dot{S}}_{m}$) to account for this misfit as follows

(4) $${\dot{S}}_{\mathrm{adjusted,m}}={\dot{S}}_{m}+\left(T_{m}-T_{i}\right)\cdot {\mathrm{TSLS}}_{m}$$

where TSLS_*m*_ is the sensitivity estimated for *m*th CMIP6 model. This is a small adjustment as the model weights ensure that the temperature misfit is small. This combination strategy ensures that the CMIP6 ensemble is weighed such that it is consistent with future temperatures used by the ice emulator and consequently AR6 as the emulator was designed to be consistent with the AR6.

The TSLS estimates are, by design, near independent of GMST and thus climate sensitivity (Grinsted & Christensen, 2021). We therefore directly combine TSLS estimates from the different contributors, where we simply combine each set of TSLS from an ice emulator sample with the steric TSLS from a random CMIP6 model.

## 4 Results and Discussion

The results and discussion are divided into subsections for each component followed by a subsection examining the integral, that is, GMSL. This is followed by a summary subsection with TSLS estimates for all components as a function of temperature. The results of each component are discussed in the relevant subsection. When TSLS ranges are included, they are quoted as the two sigma, 90th percentile range.

### 4.1 Steric

**Figure 1:**
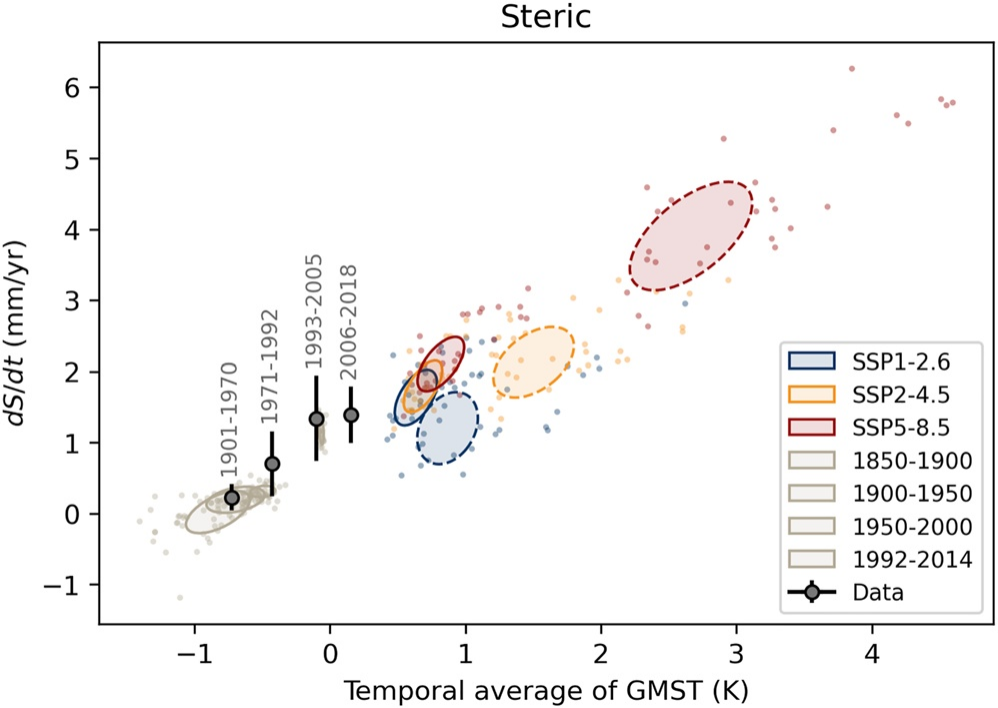
The steric contribution to sea level rise plotted against the temporal average of Global Mean Surface Temperature (GMST) for different periods. Black dots show observational estimates from AR6 with 1*σ* ranges. Colored dots show the response of the individual models in the CMIP6 archive in different periods and for different scenarios. Covariance ellipses show the 1*σ* range of the 2016–2050 (solid) and 2051–2100 (dashed), when models are weighted according to Equation 3.

Although there is significant model spread, it is evident that the assumption of a linear relationship between the averaged temperature and sea level rate for the thermosteric component is valid and that the gradient of the linear fit for CMIP6 models and the observations are broadly consistent (Figure 1). This is not surprising as thermal expansion of the oceans is a linear function of ocean heat content to first order. It is, nonetheless, reassuring that the models and observational data are broadly consistent, despite the relatively limited steric data available for the first half of the Twentieth century.

The linear regression is best constrained for the period 2051–2100 which spans the largest temperature range between scenarios. For this period, we find that models have a steric sensitivity of 1.5 ± 0.2 mm/yr/K. The median estimates for the earlier periods (1850–2015 and 2015–2050) are consistent (1.7 ± 0.5 and 2.1 ± 0.8 mm/yr/K) but show substantially more scatter as the data span a smaller temperature range and, consequently, is less well constrained. The observations indicate a sensitivity of 1.4 ± 0.5 mm/yr/K.

The observational data imply a balance temperature of −0.9 ± 0.2 K which is close to the pre‐industrial value. This suggests that to mitigate SLR in the future would require a substantial reduction in present‐day GMST, noting that internal variability has been responsible for about 5 cm of sea level change over the pre‐industrial Common Era (Kopp et al., 2016). We cannot extract a meaningful estimate of balance temperature from the models as it is necessary to apply a drift correction to CMIP6 models, as mentioned earlier. This drift correction is a vertical offset in Figure 1 which will affect the balance temperature.

### 4.2 Greenland Ice Sheet (GrIS)

**Figure 2:**
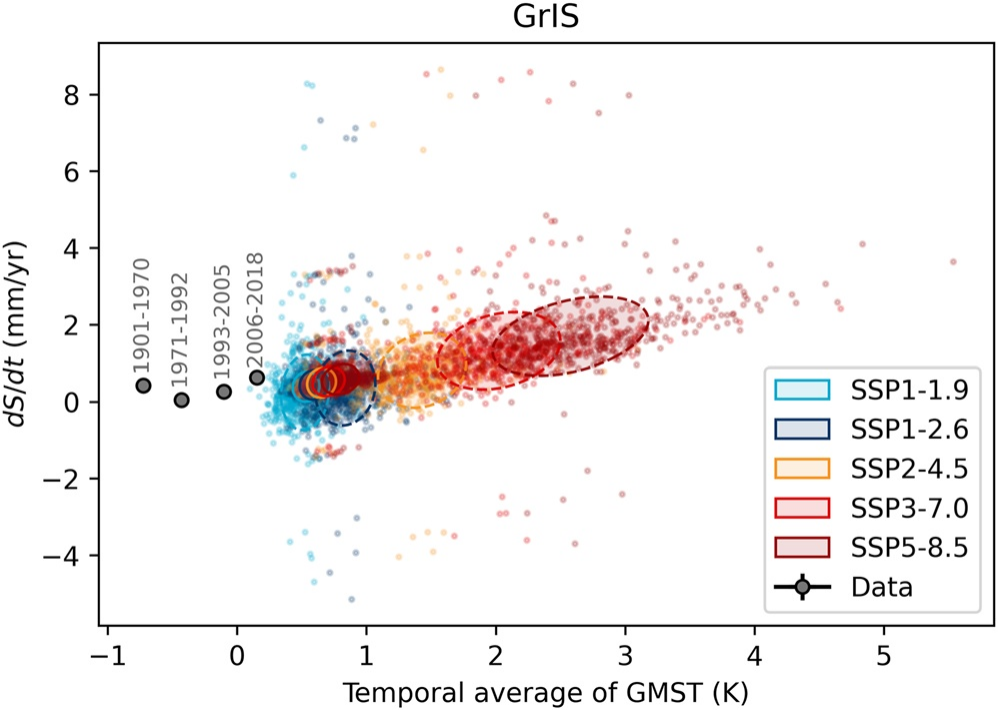
The Greenland contribution to sea level rise plotted against the temporal average of Global Mean Surface Temperature (GMST) for different periods. Black dots show observational estimates from AR6. Colored dots show the response of 500 individual models (Edwards et al., 2021) in two different periods for five scenarios. Covariance ellipses show the 1*σ* range of the 2016–2050 (solid) and 2051–2100 (dashed).

**Figure 3:**
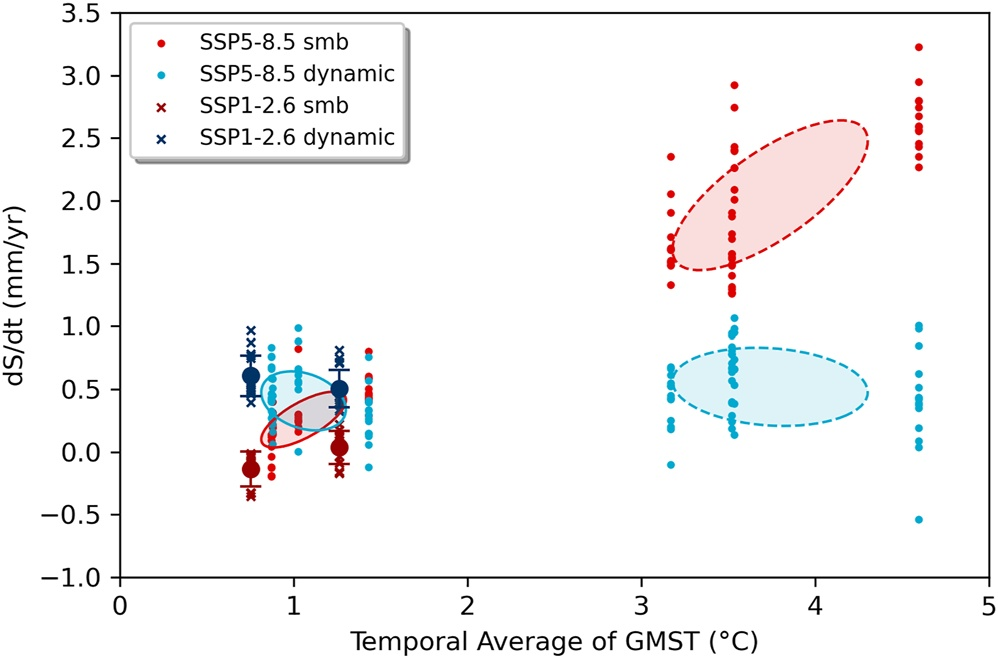
The Greenland contribution to sea level rise from SMB and dynamics plotted against the temporal average of Global Mean Surface Temperature (GMST) of CMIP6 models used in ISMIP6 (Payne et al., 2021). Covariance ellipses show the 1*σ* range of the 2016–2050 (solid) and 2051–2100 (dashed) periods. For SSP1‐2.6 only one CMIP6 model was used, so the 1*σ* range is shown by vertical error bars.

Figure 2 indicates a near‐linear sea level trend for the GrIS versus average temperature (2051–2100: 0.8 ± 0.2 mm/yr/K), which, as discussed below, is likely a result of mass loss being dominated by SMB over ice dynamics in the models. In a subset of ISMIP6 model simulations it is possible to separate the role of these two components of mass loss. We do this using the results presented in (Payne et al., 2021), and find that the dynamic component of mass loss is both limited and insensitive to average temperature, whereas the SMB component has a positive linear relationship with GMST (Figure 3). As a result, SMB becomes an increasingly dominant component of mass loss under SSP5‐8.5, from being approximately equal to the dynamic component in the earlier part of the century, to being a factor of 2–3 greater in the second half. The ISMIP6 simulations apply a SMB‐elevation feedback, such that melt increases as elevation decreases (Nowicki et al., 2020). This acts to enhance SMB losses with surface lowering, contributing to the increasing dominance of this component over the century.

The way the future ocean forcing was applied to the ice sheet models means that the projections are unlikely to exhibit a non‐linear dynamic response over the next century. The majority of the ISMIP6 GrIS model projections use a parameterization based on a simple linear function that determines a change in terminus position given subglacial discharge (estimated from modeled surface runoff) and ocean thermal forcing (Slater et al., 2019). The sector‐averaged strength of the empirical relationship is used to determine terminus positions of each marine terminating glacier in the future, given projected subglacial discharge and ocean thermal forcing, which are then applied as a mask to the modeled ice sheet extent. As a result of this simple approach, non‐linearities in future dynamic behavior, for example, due to local glacier and bedrock geometry, are not directly accounted for in the standard Greenland projections. In contrast (Choi et al., 2021), use a calibrated calving law in their Greenland simulations and find that the regions dominated by marine terminating glaciers in Northern Greenland exhibit a stronger dynamic response over the twenty‐first Century, compared to the ISMIP6 projections that use the empirical retreat parameterization. We infer from this, and our results that the dynamic response obtained in the ISMIP6 simulations is too weak.

The TSLS derived from the GrIS projections are consistent with the historic data since 1971. The 1901–1970 estimate appears anomalous compared to later periods. We see several possible explanations for this anomaly. The early estimate was derived from trimline elevations associated with the Little Ice Age maximum extent of the ice sheet (Kjeldsen et al., 2015). However, trimlines only provide observational constraints on changes in marginal geometry. In reality it is possible that interior mass gains could have partially offset marginal losses, which seems conceivable considering this pattern is seen in present‐day satellite observations (e.g., Helm et al., 2014). Further, the Little Ice Age maximum does not have a well defined end date, and period length influences the estimated rate. One explanation could therefore be that the 1901–1970 rate is biased high because there are no observations that constrain interior mass change, or due to uncertainty in timing. Additionally, the ice sheet responds to local climate change, which in turn is linked to global climate. The ice sheet response at the end of the Little Ice Age is linked to a change in regional air temperature (Box, 2013; Box & Colgan, 2013), which is not captured by changes in GMST unless it is a global effect.

### 4.3 West Antarctic Ice Sheet (WAIS)

**Figure 4:**
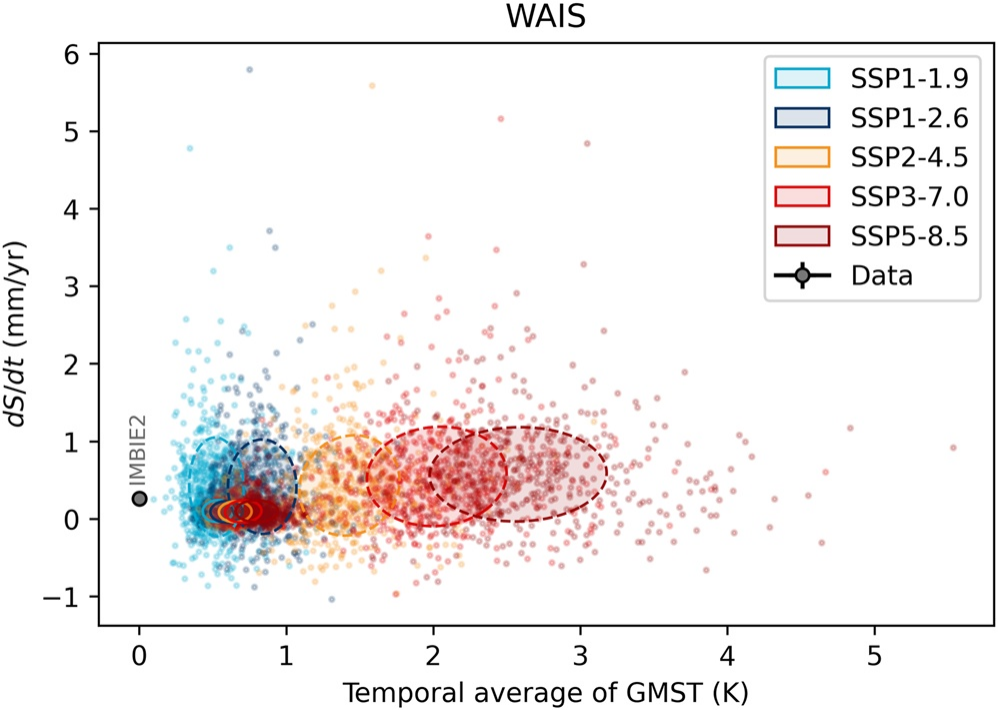
The West Antarctic Ice Sheets (WAIS) contribution to sea level rise plotted against the temporal average of Global Mean Surface Temperature (GMST) for different periods. Black dot shows observational estimates from IMBIE2 (Shepherd et al., 2018). Colored dots show the response of 500 individual models (Edwards et al., 2021) in two different periods for five scenarios. Covariance ellipses show the 1*σ* range of the 2016–2050 (solid) and 2051–2100 (dashed).

**Figure 5:**
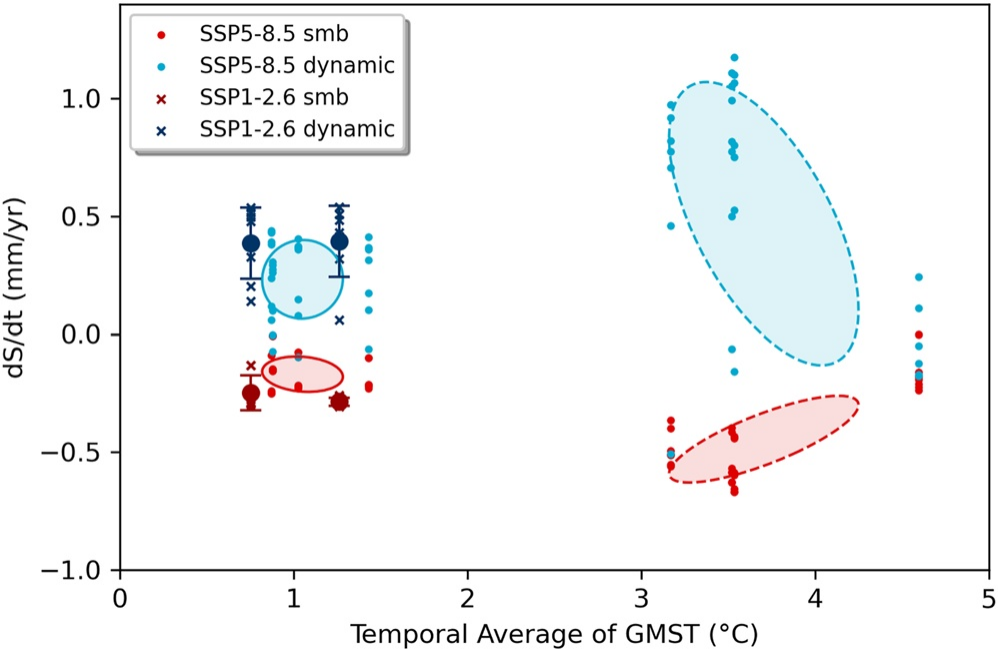
The West Antarctic Ice Sheets (WAIS) contribution to sea level rise from surface mass balance (SMB) and dynamics plotted against the temporal average of Global Mean Surface Temperature (GMST) of CMIP6 models used in ISMIP6 (Payne et al., 2021). Covariance ellipses show the 1*σ* range of the 2016–2050 (solid) and 2051–2100 (dashed) periods. For SSP1‐2.6 only one CMIP6 model was used, so the 1*σ* range is shown by vertical error bars.

The WAIS shows no clear sensitivity to average temperature, which suggests that the marine ice sheet instability is not initiated, or is not of sufficient amplitude, during the twenty‐first century for any of the climate forcing scenarios including SSP5‐8.5 (Figure 4). It is worth noting that this scenario has an averaged global temperature anomaly of +2.6 K over the latter half of the twenty‐first century, but which is amplified at high latitudes. The lack of scenario dependence in mass loss through ice dynamics is demonstrated by the subset of ISMIP6 simulations shown in Figure 5. These results also suggest that the timescale for the emergence of dependence on scenario in dynamic processes extends beyond the current century (Lowry et al., 2021).

**Figure 6:**
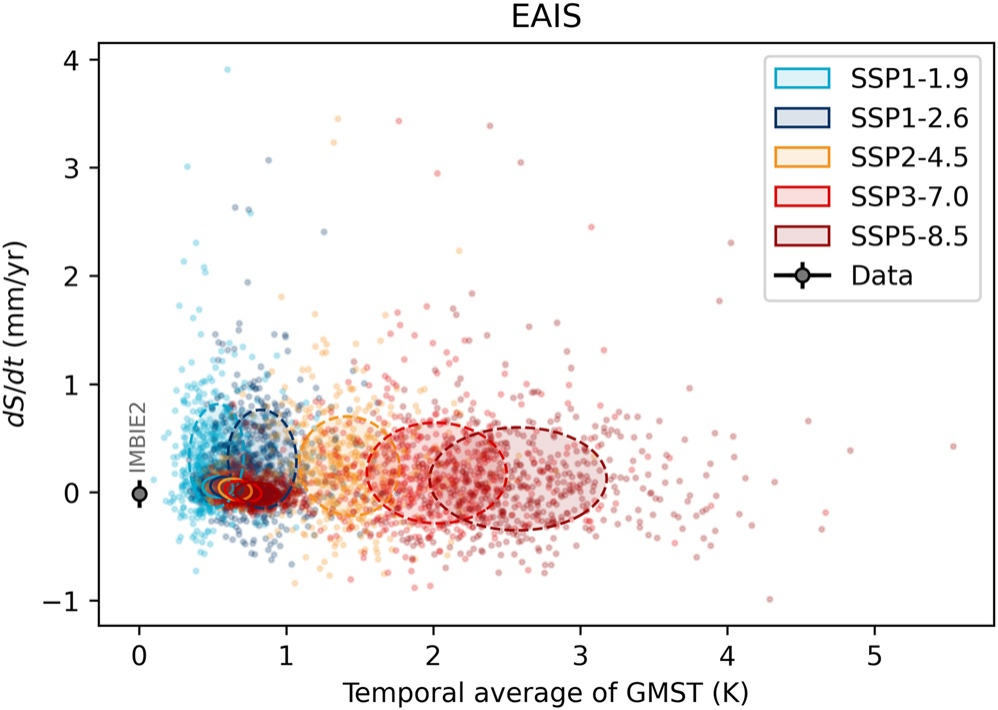
The East Antarctic Ice Sheets (EAIS) contribution to sea level rise plotted against the temporal average of Global Mean Surface Temperature (GMST) for different periods. Black dot shows observational estimates from IMBIE2 (Shepherd et al., 2018). Colored dots show the response of 500 individual models (Edwards et al., 2021) in two different periods for five scenarios. Covariance ellipses show the 1*σ* range of the 2016–2050 (solid) and 2051–2100 (dashed).

WAIS mass loss is dominated by ice dynamic processes with no dependence on scenario. Conversely, for most models, SMB shows increasingly positive trends (negative sea level contribution) with increasing global average temperature throughout Antarctica, which agrees with previous independent modeling studies even going back as far as the fourth IPCC assessment report in 2007 (Gregory & Huybrechts, 2006; Lenaerts et al., 2016). However, there is an indication that at higher temperatures this relationship could switch, where instead of increasing, the SMB starts to decrease (and contribute positively to sea level) as global temperatures rise further. This is similar to what is observed over the Antarctic Peninsula, where increased runoff over the ice shelves will contribute to a decrease in SMB (Kittel et al., 2021), in addition to the formation of surface lakes contributing to increased melting (Buzzard et al., 2018). This could have a knock‐on impact on the ice dynamic contribution, through the reduction in buttressing of upstream grounded ice (Fox‐Kemper et al., 2021).

### 4.4 East Antarctic Ice Sheet (EAIS)

**Figure 7:**
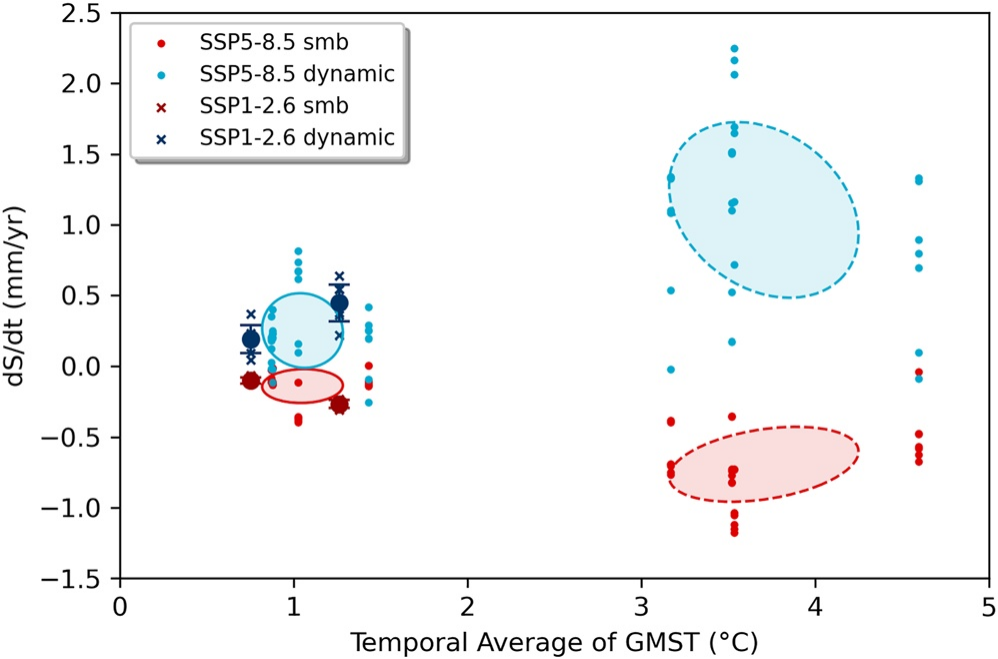
The East Antarctic Ice Sheets (EAIS) contribution to sea level rise from surface mass balance (SMB) and dynamics plotted against the temporal average of Global Mean Surface Temperature (GMST) of CMIP6 models used in ISMIP6 (Payne et al., 2021). Covariance ellipses show the 1*σ* range of the 2016–2050 (solid) and 2051–2100 (dashed) periods. For SSP1‐2.6 only one CMIP6 model was used, so the 1*σ* range is shown by vertical error bars.

The rate of sea level contribution from the EAIS has a weak negative relationship with GMST (2051–2100: −0.1 ± 0.2 mm/yr/K), indicating that increases in accumulation under warmer conditions outweigh any losses from other processes. In fact, as was the case for the WAIS, ice dynamics shows no obvious trend with temperature (Figure 7) suggesting that the only process relevant during the twenty‐first century, irrespective of SSP, is changes in snowfall. This conclusion is consistent throughout the IPCC assessment reports (Church et al., 2013; Gregory & Huybrechts, 2006) despite numerous advances and developments in process understanding, model resolution and numerics. However, the lack of sensitivity is likely more to do with how the forcing is prescribed and defined than with the fidelity of the ice sheet models. Taking a different approach by assessing the response to sub‐shelf melting, a separate modeling study found a significant sea level contribution was obtained for the EAIS with a discernible sensitivity to the forcing scenario (Levermann et al., 2020).

The spread in dynamic response to global mean temperature in the EAIS is greater than in the WAIS from 2051 to 2100, especially for the highest global mean temperatures (Figure 7). However, the ice sheet model simulations at the high end of the temperature range have all been forced with a single CMIP6 model, and the spatial pattern of warming can vary substantially between models. As with WAIS, the EAIS SMB remains positive (negative sea level contribution) across all simulations driven by the available CMIP6 models and future scenarios.

For both EAIS and WAIS, the early twenty‐first century time period tends to produce lower rates of sea level contribution, with a smaller spread, than the late twenty‐first century time period for a comparable temperature change. This is indicative of the diverging responses of the ice sheet model ensemble members over time, as some continue with a linear trend in the sea level contribution for the duration of the century, whereas others, particularly at the high tail end of the distribution have a super‐linear response (Seroussi et al., 2020).

### 4.5 Glaciers and Ice Caps (GIC)

**Figure 8:**
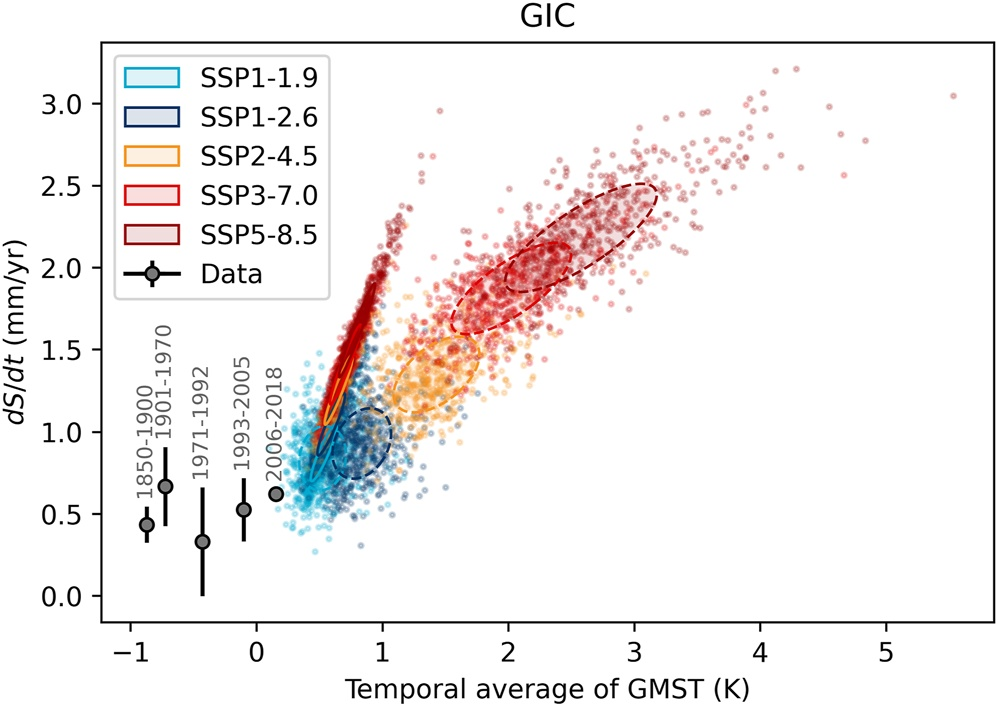
The glaciers and ice caps (GIC) contribution to sea level rise plotted against the temporal average of Global Mean Surface Temperature (GMST) for different periods. Black dot shows observational estimates from AR6. Colored dots show the response of 500 individual models (Edwards et al., 2021) in two different periods for five scenarios. Covariance ellipses show the 1*σ* range of the 2016–2050 (solid) and 2051–2100 (dashed).

GIC display a bimodal behavior dependent on the time period (Figure 8). The TSLS is higher for the first half of the twenty‐first century because, as they melt, the GIC area declines and hence has less potential to contribute mass to sea‐level change. This reduces their sensitivity to further temperature increases in the second half of the century. Nonetheless, over a 50 year period a linear sensitivity to average temperature change is a reasonable approximation, but over longer time periods the sensitivity changes. This may also explain why the observational records do not fall on a straight line, although another reason for that is likely due to the disappearance of small glaciers during the twentieth century and missing glaciers from the global inventory that are below a minimum size threshold (Parkes & Marzeion, 2018). The anomalous early twentieth century records could also be partly due to the post Little Ice Age response of GIC. The Little Ice Age was predominantly a Northern Hemisphere signal, which is also where the GIC are mostly situated, rather than a global mean temperature anomaly. This regional versus global difference could result in the 1901–1970 sea level contribution biasing high (see also for the GrIS). Post‐2100, we would expect the TSLS to continue to decrease in line with diminishing glacier area. We note, however, that a recent study using a deep learning approach and comparing a model with a linear response to climate forcing (as used in, e.g., most GlacierMIP and hence AR6 simulations) with a nonlinear version found that linear models tend to overestimate the response for the high end scenarios in particular but also for glaciers with a longer response time (Bolibar et al., 2022). This finding implies that the TSLS is likely too high when assuming a linear response. If correct, this would result in the twenty‐first century TSLS lying closer to the historic value.

### 4.6 Global Mean Sea Level (GMSL)

**Figure 9:**
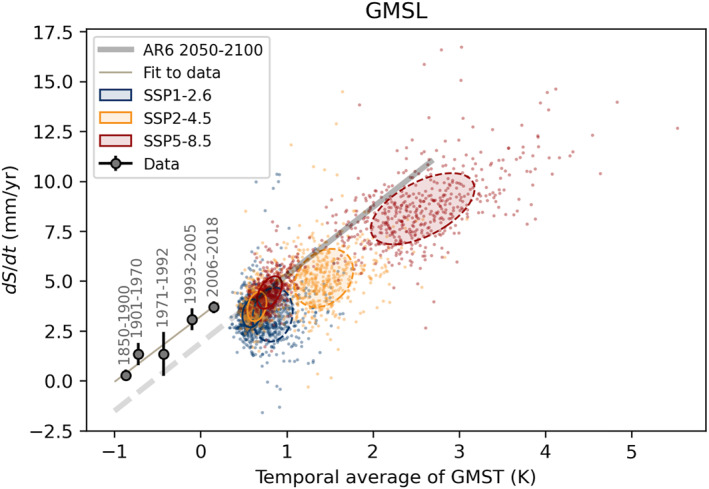
The rate of Global Mean Sea Level rise plotted against the temporal average of Global Mean Surface Temperature (GMST) for different periods. Black dots show observational estimates (Fox‐Kemper et al., 2021; Kemp et al., 2018), with a linear fit shown as a thin line. Colored dots show the response of 500 individual models of the contribution from land ice (Edwards et al., 2021) combined steric projections from the CMIP6 using the weighing strategy in Equation 1. Covariance ellipses show the 1*σ* range of the 2016–2050 (solid) and 2051–2100 (dashed). Light gray line shows the central projections from AR6.

We construct an ensemble of GMSL projections by combining the ice emulator projections with the steric contribution from a corresponding CMIP6 model using the selection probability weights described in Equation 3. The resulting ensemble of 500 GMSL projections is plotted in Figure 9. Both observations and projections show a near linear relationship between temperature and the rate of SLR. The TSLS slope for 2016–2050 is 5.3 ± 1.0 mm/yr/K, which is greater than the 3.0 ± 0.4 mm/yr/K estimated for 2051–2100. Observations indicate a sensitivity of 3.3 ± 0.4 mm/yr/K. The central estimate of the sensitivity derived from the AR6 projections during the latter half of the twenty‐first century is also 3.4 mm/yr/K. This is significantly larger than that obtained in the AR5 (2.7 mm/yr/K) (Grinsted & Christensen, 2021), which is likely due to the increased climate sensitivity of CMIP6 models (Forster et al., 2020). The observational estimates of TSLS determined here and in (Grinsted & Christensen, 2021) are not identical because different data were used. In particular, the AR6 assessed sea level rate for 1901–1990 (1.35 ± 0.35 mm/yr) is greater than the rate in Grinsted and Christensen (2021) (1.1 ± 0.3 mm/yr; Dangendorf et al., 2017). This greater rate has the effect that the acceleration into the satellite altimetry era appears smaller.

The model projections appear vertically offset from the relationship indicated by the observational data with AR6 being about 1 mm/yr below the empirical relationship and the ensemble falling ∼2 mm/yr lower (Figure 9). This suggests that model rates are biased low by a constant amount. This is not surprising as the emulated land ice contributions have not been adjusted for drift. The emulated ice sheet contributions are based on the ISMIP6 which by design results in zero trend for present‐day temperatures. In addition, here we are not including small quasi‐constant components of the sea level budget such as the effect of ocean bottom deformation, the deep steric term or land water storage. These terms, while important for closing the sea level budget, have a negligible effect on the TSLS (Vishwakarma et al., 2020).

The intercept of the GMSL TSLS relationship on the *x*‐axis we term the balance temperature and represents the value at which SLR is zero. This value is important for considering attribution of SLR. A reconstruction of sea level over the last 2,500 years shows variations around a mean close to zero up until the start of the twentieth century (Kopp et al., 2016) and appears closely tied to global temperature anomalies. Internal variability in the climate system accounts for around 7 cm of sea level variation over the pre‐industrial period. Our results are consistent with this, indicating that the balance temperature of −1.0 ± 0.1°C is equivalent to pre‐industrial temperature. In other words, SLR since the start of the twentieth century can be attributed to anthropogenic global warming plus a much smaller component (circa ±7 cm) due to internal variability. This interpretation is supported by a recent reassessment of the contribution of anthropogenic warming versus the post Little Ice Age response of GIC during the twentieth century (Roe et al., 2021), where they conclude that the response is entirely driven by anthropogenic warming. This conclusion has implications for attribution studies that assumed that early twentieth century SLR was largely due to a GIC response to the Little Ice Age (Slangen et al., 2016). In that study, they infer that natural forcing and internal variability contribute about 10% and 35% to SLR for 1970–2005 and 1900–2005, respectively. The revised attribution of GIC means that twentieth century SLR is dominated by anthropogenic global warming with about 10% due to internal variability and natural forcing for the whole century (Roe et al., 2021; Slangen et al., 2016). Our results are consistent with this.

### 4.7 Summary of Sensitivities

**Figure 10:**
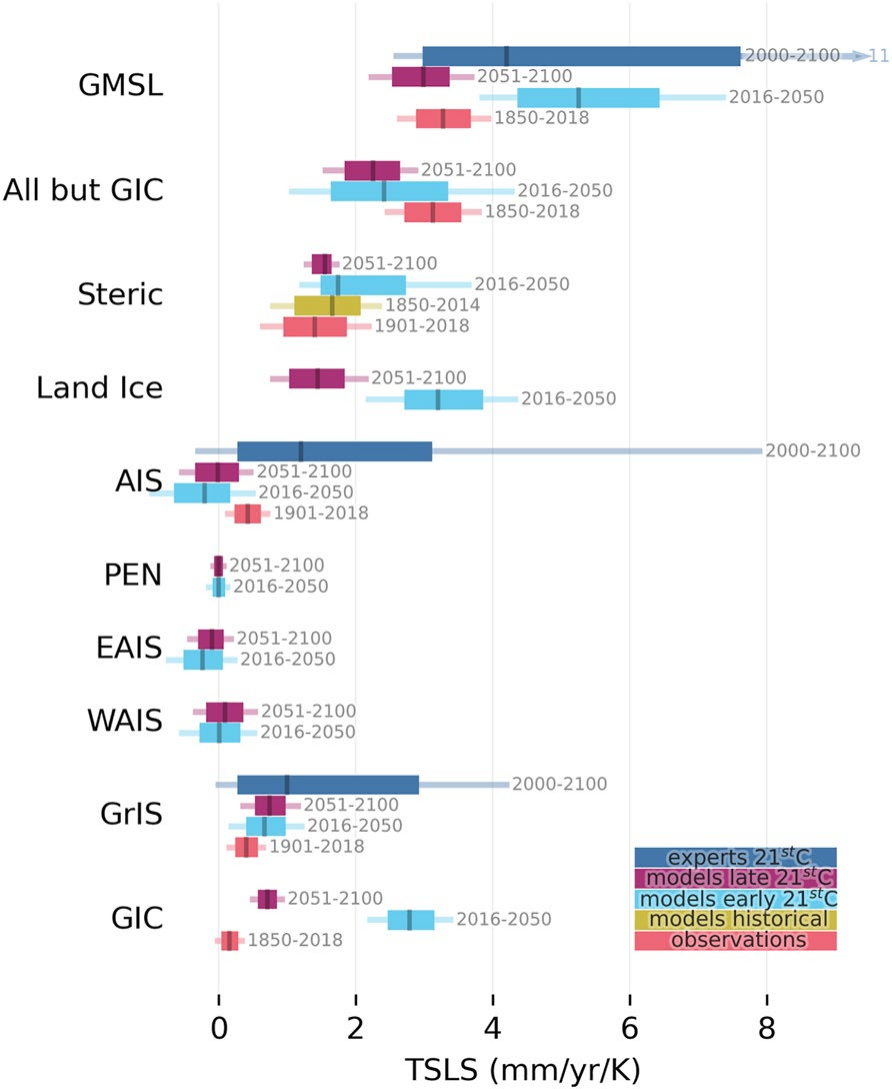
Estimated transient sea level sensitivity (TSLS) for different contributors and different periods. Uncertainty ranges are shown as 5%–17%–50%–83%–95%.

We summarize the range of estimated TSLS for every model of the major contributors in Figure 10. Most contributors to SLR appear to have a near time‐invariant TSLS with GIC being a notable exception. This is in accordance with expectations, as the ice sheets and ocean heat content have multi‐centennial response times, and the response on the time scales considered in this paper can therefore be considered transient. Glaciers, however, have a much shorter response time, and thus GIC has started to equilibrate to the new warmer climate by the end of a century, and we see that as a reduced TSLS in the late twenty‐first century. This reduces the universality of the TSLS metric for the GIC contribution over a century time scale.

The fast GIC response directly affects the GMSL behavior, which results in TSLS changing between periods. Historical observations indicate a GMSL sensitivity of 3.3 ± 0.4 mm/yr/K. Model projections indicate a greater sensitivity prior to 2050 (5.3 ± 1.0 mm/yr/K), but smaller sensitivity post 2050 (3.0 ± 0.4 mm/yr/K). This is in line with the central TSLS estimate derived from expert assessments for the entire twenty‐first century (+4.2 mm/yr/K [90%: 2.6–11 mm/y/K]) (Bamber et al., 2019). The deviations between these estimates has clearly largely been mirrored from the GIC response (Figure 10), which masks the more stationary TSLS of the remainder of the sea level budget. We therefore also report the sensitivity of GMSL minus GIC (labeled “All but GIC” in Figure 10). This reveals a discrepancy between the sensitivity of models and estimates from historical data. Historical observations indicate a sensitivity of 3.1 ± 0.4 mm/yr/K, which is ∼30% greater than model projections. This discrepancy is hard to justify physically as models display a stationary sensitivity over the twenty‐first century (+2.5 mm/yr/K pre‐2050 vs. +2.3 mm/yr/K post‐2050). This suggests that models of at least one of the remaining components (steric, GrIS, or AIS) underestimates the sensitivity to warming.

We find that the steric sea level contribution from CMIP6 models have a transient sensitivity (2051–2100: 1.5 ± 0.2 mm/yr/K) to warming which is marginally greater but compatible with the historical estimate (1.4 ± 0.5 mm/yr/K; see Figure 10). The steric contribution therefore cannot explain the “All but GIC” model‐data discrepancy.

Greenland ice sheet models are more sensitive to temperature change than our estimate from historical data (0.8 ± 0.2 mm/yr/K vs. 0.4 ± 0.2 mm/yr/K). This could indicate a bias in either the model or the observational estimates, or that the transient sensitivity is increasing over time. Results from a structured expert judgment (Bamber et al., 2019) imply that experts judge that the GrIS may be more sensitive (+1.0 mm/yr/K) than models and observations imply. That increase in sensitivity would require a non‐linear response to temperature that is not evident in the models (Figure 2). This suggests that experts are aware of structural uncertainties in the modeled GrIS contribution that could lead to a substantial non‐linearity in the response as implied by some recent modeling and observational studies (Aschwanden et al., 2019; King et al., 2020; Sasgen et al., 2020). We conclude, therefore, that GrIS is also unlikely to be the source for the “All but GIC” model‐data discrepancy.

Models of the AIS display negligible sensitivity to warming (Figure 10) in contrast to historical data which indicate that AIS has a sensitivity of +0.4 ± 0.2 mm/yr/K. Restricting the observational estimate to the satellite era, where the AIS contribution is better constrained, increases the estimate to +0.5 mm/yr/K. This is far short of a mean centennial value as the observational record is only about 30 years. There is some evidence, however, that part of the observed behavior of the WAIS during that period is due to a forced climate signal (Holland et al., 2019). Further, experts expect a large difference in the AIS contribution between a 2°C and a 5°C scenario (Bamber et al., 2019) which implies a transient sensitivity of +1.2 mm/yr/K. Such an increase over the historical sensitivity indicates that experts consider a non‐linearity in the AIS sensitivity to be possible. Considerable uncertainty remains regarding the role of certain processes during the twenty‐first century for the AIS (DeConto et al., 2021; Edwards et al., 2019) and this is likely reflected in the wider range of values obtained in the expert elicitation. This behavior is not reflected in the model projections, and this partly explains the “All but GIC” model‐data discrepancy. The difference between the observational and model derived sensitivity of the AIS is however insufficient to fully account for the “All but GIC” discrepancy.

The AIS contribution can be partitioned into dynamics and SMB, and regionally into EAIS, WAIS, and Peninsula. The EAIS and WAIS have been discussed in preceding sections. The dynamic contributions of both EAIS and WAIS show little scenario dependence in the ISMIP6 models and are thus relatively insensitive to warming (Figures 5 and 7). The model sensitivities of both ice sheets are therefore predominantly a result of the SMB response to warming. Warming tends to result in increased melt and runoff, but also increased accumulation due to the greater moisture holding capacity of the atmosphere. The SMB sensitivity to warming can therefore be both positive and negative. The accumulation response dominates over the EAIS which results in a net negative TSLS for the ice sheet (Figures 6 and 7). Accumulation and melt is closer to balance over the WAIS, and for the most intense warming scenarios the melt response can start to dominate the SMB sensitivity in some models (Figure 5). The net result for the WAIS is a slightly positive central estimate of the TSLS (Figure 4). We find that models of the Antarctic Peninsula have a near zero sensitivity to warming (0.00 ± 0.05 mm/yr/K). This is surprising considering that satellite observations show rapid and accelerating glacier mass loss in the region (Wouters et al., 2015). Further, a glacier modeling study found SMB in the region to be particularly sensitive to warming (Hock et al., 2009). Further, it has been suggested that the lack of scenario dependency in the modeled dynamic response of AIS over the twenty‐first century is due to inadequate understanding of ice flow and sliding, which results in high uncertainty in sea level projections and thus overlap between scenarios (Lowry et al., 2021). However, over longer time scales they find that large differences between high and low emission scenarios do emerge. This conclusion is also supported by the most recent study of the AIS response when accounting for the marine ice cliff instability (Deconto et al., 2021). This could in part explain the mismatch between our observed and modeled TSLS results.

## 5 Conclusions

We have examined how the contributions to the sea level budget relate to GMST from both models and data. We approximate AR6 model projections (Fox‐Kemper et al., 2021) by using weighted CMIP6 models and the output of an ice emulator (Edwards et al., 2021) using ISMIP6 (Goelzer et al., 2020; Seroussi et al., 2020) and GlacierMIP (Marzeion et al., 2020). We find the rate of the individual contributions to be near linear in average temperature, and quantify the slope as the TSLS. We thus focus our attention on the response sensitivity to a change in warming, rather than the total sea level contribution which is also affected by drift.

Models of all contributors, apart from GIC, show little change in TSLS over the twenty‐first century. A comparison between the historical sensitivity estimated from observations, and the sensitivity implied by model projections can therefore serve as a sanity check on the model response for most contributors. GIC shows a marked change in TSLS over the twenty‐first century (Figure 8), which is expected as many glaciers have decadal scale response times. The TSLS concept is therefore of limited utility for the GIC contribution. While GIC only contributes a fraction to GMSL, this limits how closely we should expect twenty‐first century TSLS to match the historical GMSL sensitivity (3.3 ± 0.4 mm/yr/K). We therefore also examine the residual response after removing the GIC contribution, and identify a substantial discrepancy between the sensitivity inferred from models versus historical data. The historical estimate of the “All but GIC” sensitivity (3.1 ± 0.4 mm/yr/K) is 30% greater than the model sensitivity (Figure 10). This strongly suggests that at least one of the ice sheets, or the steric contribution has an overly muted response to warming. The sensitivity of GrIS and steric show a closer correspondence with historical estimates. We find that the AIS is the most likely candidate as most models have low to negative sensitivity to warming in contrast to our historical estimate of 0.4 ± 0.2 mm/yr/K (Figure 10). We speculate the WAIS and Antarctic Peninsula to be the source of the discrepancy based on recent mass loss trends in the region (Shepherd et al., 2018; Wouters et al., 2015).

A recent study found that the ice sheet models were unable to reproduce recent observed trends in mass loss (specifically from GrIS) and argued that this raises concerns regarding model skill (Aschwanden et al., 2021). This is a separate issue from the TSLS discrepancy we identify in this paper which suggests that AIS model sensitivity is biased low. The TSLS quantifies how mass loss accelerates under warming and is unaffected by how well it captures present day trends. Modeling protocols such as removing a control run, or how the model is spun‐up influence long term trends. An imperfect initial state in a model with a long response time can result in an unforced long term model drift. Model drift is a challenging issue in all models with a very long response time, and so affects both ice sheets (Goelzer et al., 2018) and steric models (Slangen et al., 2016). A reasonable match to the present‐day rate is therefore not a sufficient validation of models of components with long response times, and vice versa. This is perhaps best illustrated by the ensemble of GrIS models (Figure 2) which is unable to match present‐day trends (Aschwanden et al., 2021; vertical offset in Figure 2), while having a TSLS that is in good agreement with historical records (Figures 2 and 10). Model intercomparison projects such as ISMIP6 and CMIP6 are crucial to assessing model skill. A limitation of the TSLS comparison in this paper is that we compare past to future response and some models were not run for the historical period. We therefore recommend that the protocol for future ice sheet model intercomparisons is inspired by CMIP6 to include historical runs starting in 1850, to enable stronger validation against data. However, this is not feasible for model initialization methods that rely on the assimilation of high‐quality ice‐sheet wide observations from satellites, for example, inverting for model parameters by matching observed velocities. It is a challenge to critically assess the sensitivity in models without a past, but TSLS comparisons to historical estimates remain a viable option.

The near‐stationary sensitivity of most contributors has practical implications for coastal planners and decision‐makers. Regional SLR projections are usually constructed by modeling the impact of the mass loss from individual contributors on the static equilibrium of the sea surface (due to e.g., gravitational redistribution of mass), and change due to dynamical sea level is then accounted for. Often it is assumed that the dynamical sea level scales with global mean steric expansion. In practice, this means that the local sea level is a weighted sum of all the individual contributors. If we explicitly account for GIC, local vertical land motion, weather and tidal variability (e.g., following Frederikse et al., 2016) then we are left with a residual that responds near linearly to warming according to models. This can potentially be leveraged to make local relative sea level projections by extrapolation. Further study is needed to assess the feasibility of this approach.

Beyond the year 2100, we expect feedbacks to play an increasing role in the ocean heat uptake and ice sheet mass loss. We therefore expect the TSLS of these contributors to start deviating from historical values and from a linear trend. Eventually the response can no longer meaningfully be considered transient, and it will be more useful to consider the equilibrium sensitivity to warming and sea level commitment (Clark et al., 2016; Fox‐Kemper et al., 2021); that is, how many meters can we ultimately expect for a given forcing? Here paleo records, rather than historical records, can serve as an important constraint for models. We note that a credible equilibrium response does not guarantee a credible transient sensitivity as the equilibrium can be approached at different speeds (Gilford et al., 2020). Finally, we associate about 90% of SLR since the start of the twentieth century with anthropogenic global warming.

## Supporting information

Supporting Information S1

## Data Availability

This paper relies on publically available data from CMIP6, ISMIP6 and Edwards et al. (2021). Derived data sets will be made available in a public repository (e.g., Zenodo) upon acceptance of the manuscript. Until then these data are available in our source code repository: https://github.com/cmip6moap/project01/tree/main/data/processed_data.
