# Supplementary material for: The Transient Sea Level Response to External Forcing in CMIP6 Models
Source: Earths Future. 2022 Oct 24;10(10):e2022EF002696. doi: 10.1029/2022EF002696 (PMC9786795; doi:10.1029/2022EF002696)

Supplementary Information:

Table S1: List of CMIP6 models and variants used in the thermosteric calculation.

| Model | Scenario | Variant(s) |
| --- | --- | --- |
| ACCESS-CM2 | Historical | r1i1p1f1 |
| ACCESS-ESM1-5 | Historical | r1i1p1f1 |
| BCC-CSM2-MR | Historical | r1i1p1f1, r2i1p1f1, r3i1p1f1 |
| BCC-ESM1 | Historical | r1i1p1f1, r2i1p1f1, r3i1p1f1 |
| CAMS-CSM1-0 | Historical | r1i1p1f1 |
| CESM2 | Historical | r2i1p1f1, r4i1p1f1, r6i1p1f1, r8i1p1f1, r11i1p1f1 |
| CIESM | Historical | r1i1p1f1 |
| CMCC-CM2-SR5 | Historical | r1i1p1f1 |
| CMCC-ESM2 | Historical | r1i1p1f1 |
| CanESM5 | Historical | r1i1p1f1, r1i1p2f1, r2i1p1f1, r2i1p2f1, r3i1p1f1, r3i1p2f1, r4i1p1f1, r4i1p2f1, r5i1p1f1, r5i1p2f1, r6i1p1f1, r6i1p2f1, r7i1p1f1, r7i1p2f1, r8i1p1f1, r8i1p2f1, r9i1p1f1, r9i1p2f1, r10i1p1f1, r10i1p2f1, r11i1p1f1, r12i1p1f1, r13i1p1f1, r14i1p1f1, r15i1p1f1, r16i1p1f1, r17i1p1f1, r18i1p1f1, r19i1p1f1, r20i1p1f1, r21i1p1f1, r22i1p1f1, r23i1p1f1, r24i1p1f1, r25i1p1f1 |
| E3SM-1-1 | Historical | r1i1p1f1 |
| E3SM-1-1-ECA | Historical | r1i1p1f1 |
| EC-Earth3-CC | Historical | r1i1p1f1 |
| EC-Earth3-Veg | Historical | r1i1p1f1 |
| FGOALS-f3-L | Historical | r1i1p1f1 |
| FGOALS-g3 | Historical | r1i1p1f1 |
| FIO-ESM-2-0 | Historical | r1i1p1f1 |
| GFDL-CM4 | Historical | r1i1p1f1 |
| GFDL-ESM4 | Historical | r1i1p1f1 |
| GISS-E2-1-G | Historical | r1i1p1f1, r2i1p1f1, r3i1p1f1, r4i1p1f1, r5i1p1f1, r6i1p1f1, r7i1p1f1, r8i1p1f1, r9i1p1f1, r10i1p1f1 |
| GISS-E2-1-G-CC | Historical | r1i1p1f1 |
| HadGEM3-GC31-LL | Historical | r1i1p1f3, r2i1p1f3, r3i1p1f3, r4i1p1f3, r5i1p1f3 |
| HadGEM3-GC31-MM | Historical | r1i1p1f3, r2i1p1f3, r3i1p1f3, r4i1p1f3 |
| INM-CM4-8 | Historical | r1i1p1f1 |
| INM-CM5-0 | Historical | r1i1p1f1 |
| IPSL-CM5A2-INCA | Historical | r1i1p1f1 |
| IPSL-CM6A-LR | Historical | r1i1p1f1, r2i1p1f1, r3i1p1f1, r4i1p1f1, r5i1p1f1, r6i1p1f1, r7i1p1f1, r8i1p1f1, r9i1p1f1 |
| IPSL-CM6A-LR-INCA | Historical | r1i1p1f1 |
| MCM-UA-1-0 | Historical | r1i1p1f1 |
| MIROC6 | Historical | r1i1p1f1 |
| MPI-ESM-1-2-HAM | Historical | r1i1p1f1 |
| MPI-ESM1-2-HR | Historical | r1i1p1f1 |
| MPI-ESM1-2-LR | Historical | r1i1p1f1 |
| MRI-ESM2-0 | Historical | r1i1p1f1 |
| NESM3 | Historical | r1i1p1f1 |
| NorESM2-LM | Historical | r1i1p1f1 |
| NorESM2-MM | Historical | r1i1p1f1 |
| SAM0-UNICON | Historical | r1i1p1f1 |
| UKESM1-0-LL | Historical | r1i1p1f2, r2i1p1f2, r3i1p1f2, r4i1p1f2, r5i1p1f3, r6i1p1f3, r7i1p1f3, r8i1p1f2, r9i1p1f2, r10i1p1f2, r11i1p1f2, r12i1p1f2, r13i1p1f2, r16i1p1f2, r17i1p1f2, r18i1p1f2, r19i1p1f2 |
| ACCESS-CM2 | SSP1-2.6 | r1i1p1f1, r2i1p1f1, r3i1p1f1 |
| ACCESS-ESM1-5 | SSP1-2.6 | r1i1p1f1, r2i1p1f1, r3i1p1f1 |
| BCC-CSM2-MR | SSP1-2.6 | r1i1p1f1 |
| CAMS-CSM1-0 | SSP1-2.6 | r1i1p1f1, r2i1p1f1 |
| CIESM | SSP1-2.6 | r1i1p1f1 |
| CMCC-ESM2 | SSP1-2.6 | r1i1p1f1 |
| CanESM5 | SSP1-2.6 | r1i1p1f1, r1i1p2f1, r2i1p1f1, r2i1p2f1, r3i1p1f1, r3i1p2f1, r4i1p1f1, r4i1p2f1, r5i1p1f1, r5i1p2f1, r6i1p1f1, r6i1p2f1, r7i1p1f1, r7i1p2f1, r8i1p1f1, r8i1p2f1, r9i1p1f1, r9i1p2f1, r10i1p1f1, r10i1p2f1 |
| EC-Earth3-Veg | SSP1-2.6 | r1i1p1f1 |
| FGOALS-f3-L | SSP1-2.6 | r1i1p1f1 |
| FGOALS-g3 | SSP1-2.6 | r1i1p1f1, r2i1p1f1, r3i1p1f1 |
| FIO-ESM-2-0 | SSP1-2.6 | r1i1p1f1, r2i1p1f1, r3i1p1f1 |
| GFDL-ESM4 | SSP1-2.6 | r1i1p1f1 |
| HadGEM3-GC31-LL | SSP1-2.6 | r1i1p1f3 |
| HadGEM3-GC31-MM | SSP1-2.6 | r1i1p1f3 |
| INM-CM4-8 | SSP1-2.6 | r1i1p1f1 |
| INM-CM5-0 | SSP1-2.6 | r1i1p1f1 |
| IPSL-CM5A2-INCA | SSP1-2.6 | r1i1p1f1 |
| IPSL-CM6A-LR | SSP1-2.6 | r1i1p1f1, r2i1p1f1, r3i1p1f1 |
| MIROC6 | SSP1-2.6 | r1i1p1f1, r2i1p1f1, r3i1p1f1 |
| MPI-ESM1-2-HR | SSP1-2.6 | r1i1p1f1, r2i1p1f1 |
| MPI-ESM1-2-LR | SSP1-2.6 | r1i1p1f1, r2i1p1f1, r3i1p1f1 |
| MRI-ESM2-0 | SSP1-2.6 | r1i1p1f1 |
| NESM3 | SSP1-2.6 | r1i1p1f1 |
| NorESM2-LM | SSP1-2.6 | r1i1p1f1 |
| NorESM2-MM | SSP1-2.6 | r1i1p1f1 |
| UKESM1-0-LL | SSP1-2.6 | r1i1p1f2, r2i1p1f2, r3i1p1f2, r4i1p1f2, r8i1p1f2, r9i1p1f2, r10i1p1f2, r11i1p1f2, r12i1p1f2, r16i1p1f2, r17i1p1f2, r18i1p1f2, r19i1p1f2 |
| ACCESS-CM2 | SSP2-4.5 | r1i1p1f1, r2i1p1f1, r3i1p1f1 |
| ACCESS-ESM1-5 | SSP2-4.5 | r1i1p1f1, r2i1p1f1, r3i1p1f1 |
| BCC-CSM2-MR | SSP2-4.5 | r1i1p1f1 |
| CAMS-CSM1-0 | SSP2-4.5 | r1i1p1f1, r2i1p1f1 |
| CIESM | SSP2-4.5 | r1i1p1f1 |
| CMCC-CM2-SR5 | SSP2-4.5 | r1i1p1f1 |
| CMCC-ESM2 | SSP2-4.5 | r1i1p1f1 |
| CanESM5 | SSP2-4.5 | r1i1p1f1, r1i1p2f1, r2i1p1f1, r2i1p2f1, r3i1p1f1, r3i1p2f1, r4i1p1f1, r4i1p2f1, r5i1p1f1, r5i1p2f1, r6i1p1f1, r6i1p2f1, r7i1p1f1, r7i1p2f1, r8i1p1f1, r8i1p2f1, r9i1p1f1, r9i1p2f1, r10i1p1f1, r10i1p2f1 |
| EC-Earth3-CC | SSP2-4.5 | r1i1p1f1 |
| EC-Earth3-Veg | SSP2-4.5 | r1i1p1f1 |
| FGOALS-g3 | SSP2-4.5 | r1i1p1f1 |
| FIO-ESM-2-0 | SSP2-4.5 | r1i1p1f1 |
| GFDL-CM4 | SSP2-4.5 | r1i1p1f1 |
| GFDL-ESM4 | SSP2-4.5 | r1i1p1f1 |
| HadGEM3-GC31-LL | SSP2-4.5 | r1i1p1f3 |
| INM-CM4-8 | SSP2-4.5 | r1i1p1f1 |
| INM-CM5-0 | SSP2-4.5 | r1i1p1f1 |
| IPSL-CM6A-LR | SSP2-4.5 | r1i1p1f1, r2i1p1f1, r3i1p1f1 |
| MIROC6 | SSP2-4.5 | r1i1p1f1, r2i1p1f1, r3i1p1f1 |
| MPI-ESM1-2-HR | SSP2-4.5 | r1i1p1f1 |
| MPI-ESM1-2-LR | SSP2-4.5 | r1i1p1f1, r2i1p1f1, r3i1p1f1 |
| MRI-ESM2-0 | SSP2-4.5 | r1i1p1f1 |
| NESM3 | SSP2-4.5 | r1i1p1f1 |
| NorESM2-LM | SSP2-4.5 | r1i1p1f1, r2i1p1f1, r3i1p1f1 |
| NorESM2-MM | SSP2-4.5 | r1i1p1f1 |
| UKESM1-0-LL | SSP2-4.5 | r1i1p1f2, r2i1p1f2, r3i1p1f2, r4i1p1f2, r8i1p1f2 |
| ACCESS-CM2 | SSP5-8.5 | r1i1p1f1, r2i1p1f1, r3i1p1f1 |
| ACCESS-ESM1-5 | SSP5-8.5 | r1i1p1f1, r2i1p1f1, r3i1p1f1 |
| BCC-CSM2-MR | SSP5-8.5 | r1i1p1f1 |
| CAMS-CSM1-0 | SSP5-8.5 | r1i1p1f1, r2i1p1f1 |
| CIESM | SSP5-8.5 | r1i1p1f1 |
| CMCC-CM2-SR5 | SSP5-8.5 | r1i1p1f1 |
| CMCC-ESM2 | SSP5-8.5 | r1i1p1f1 |
| CanESM5 | SSP5-8.5 | r1i1p1f1, r1i1p2f1, r2i1p1f1, r2i1p2f1, r3i1p1f1, r3i1p2f1, r4i1p1f1, r4i1p2f1, r5i1p1f1, r5i1p2f1, r6i1p2f1, r7i1p2f1, r8i1p1f1, r8i1p2f1, r9i1p2f1, r10i1p2f1 |
| EC-Earth3-CC | SSP5-8.5 | r1i1p1f1 |
| EC-Earth3-Veg | SSP5-8.5 | r1i1p1f1 |
| FGOALS-f3-L | SSP5-8.5 | r1i1p1f1 |
| FGOALS-g3 | SSP5-8.5 | r1i1p1f1 |
| FIO-ESM-2-0 | SSP5-8.5 | r1i1p1f1 |
| GFDL-CM4 | SSP5-8.5 | r1i1p1f1 |
| GFDL-ESM4 | SSP5-8.5 | r1i1p1f1 |
| HadGEM3-GC31-LL | SSP5-8.5 | r1i1p1f3, r2i1p1f3, r3i1p1f3, r4i1p1f3 |
| HadGEM3-GC31-MM | SSP5-8.5 | r1i1p1f3, r2i1p1f3, r3i1p1f3, r4i1p1f3 |
| INM-CM4-8 | SSP5-8.5 | r1i1p1f1 |
| INM-CM5-0 | SSP5-8.5 | r1i1p1f1 |
| IPSL-CM6A-LR | SSP5-8.5 | r1i1p1f1, r2i1p1f1, r3i1p1f1 |
| MIROC6 | SSP5-8.5 | r1i1p1f1, r2i1p1f1, r3i1p1f1 |
| MPI-ESM1-2-HR | SSP5-8.5 | r1i1p1f1 |
| MPI-ESM1-2-LR | SSP5-8.5 | r1i1p1f1, r2i1p1f1, r3i1p1f1 |
| MRI-ESM2-0 | SSP5-8.5 | r1i1p1f1 |
| NESM3 | SSP5-8.5 | r1i1p1f1 |
| NorESM2-LM | SSP5-8.5 | r1i1p1f1 |
| NorESM2-MM | SSP5-8.5 | r1i1p1f1 |
| UKESM1-0-LL | SSP5-8.5 | r1i1p1f2, r2i1p1f2, r3i1p1f2, r4i1p1f2, r8i1p1f2 |

**Ice Sheet SMB vs Dynamic contribution**

Greenland: (purple is dynamic, teal is SMB)


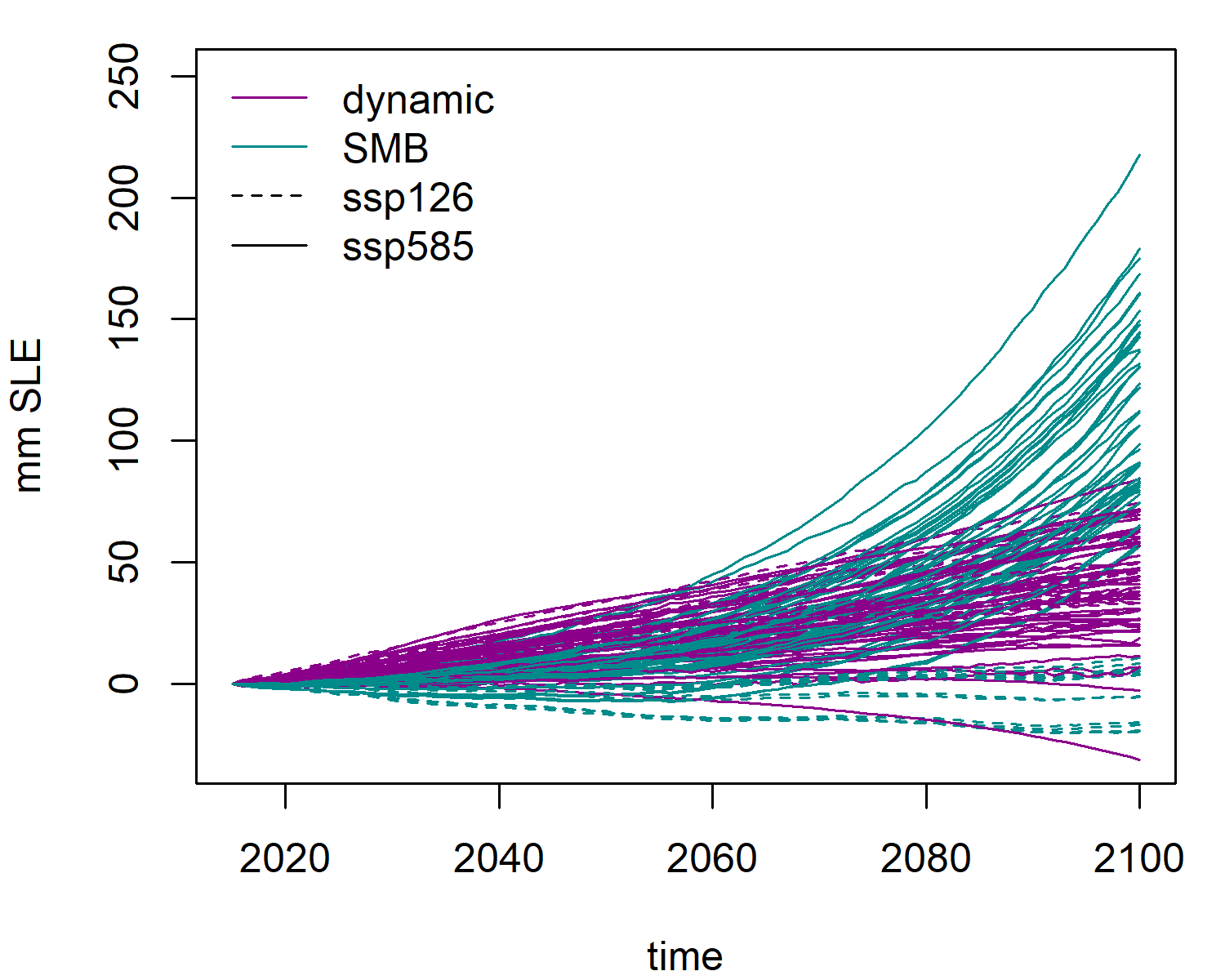


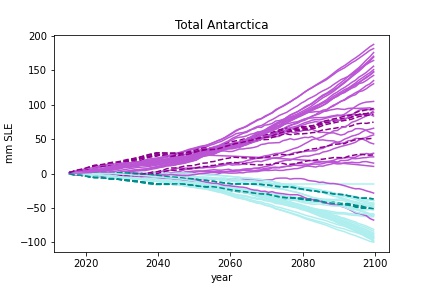

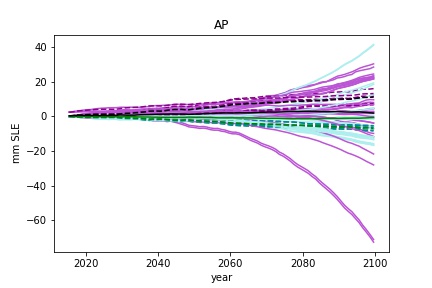

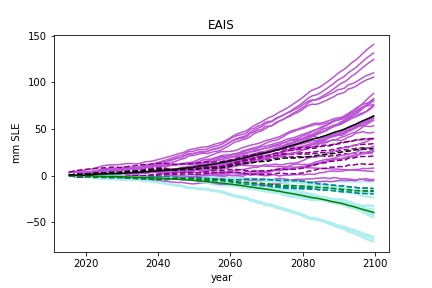

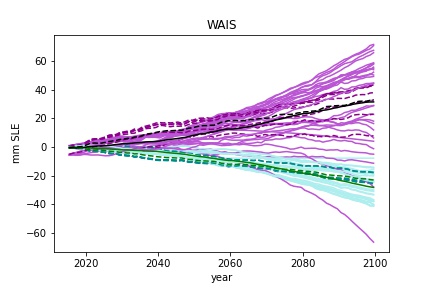

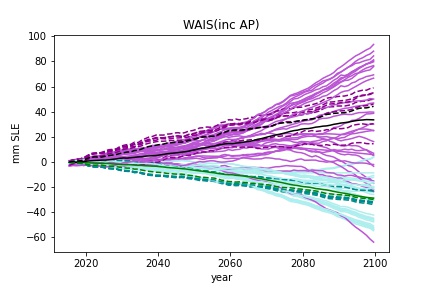

Supplement: Supplementary file 1 — Supporting Information S1 [file EFT2-10-e2022EF002696-s001.docx]
